# Non-Melanoma Skin Cancer: A Genetic Update and Future Perspectives

**DOI:** 10.3390/cancers14102371

**Published:** 2022-05-11

**Authors:** Marianela Zambrano-Román, Jorge R. Padilla-Gutiérrez, Yeminia Valle, José F. Muñoz-Valle, Emmanuel Valdés-Alvarado

**Affiliations:** 1 Centro Universitario de Ciencias de la Salud, Instituto de Investigación en Ciencias Biomédicas (IICB), Universidad de Guadalajara, Guadalajara 44340, Mexico; marianela.zambrano3682@alumnos.udg.mx (M.Z.-R.); ramon.padilla@academicos.udg.mx (J.R.P.-G.); yeminia.valle@academicos.udg.mx (Y.V.); drjosefranciscomv@cucs.udg.mx (J.F.M.-V.); 2 Doctorado en Genética Humana, Departamento de Biología Molecular y Genómica, Universidad de Guadalajara, Guadalajara 44340, Mexico

**Keywords:** skin cancer, non-melanoma skin cancer, basal cell carcinoma, squamous cell carcinoma, UVB radiation, NMSC genetics, epigenetics, tumor microenvironment, immunotherapy

## Abstract

**Simple Summary:**

Non-melanoma skin cancer (NMSC) is the main type of cancer in the Caucasian population, and the number of cases continues to rise. Research mostly focuses on clinical characteristics analysis, but genetic features are crucial to malignancies’ establishment and advance. We aim to explore the genetic basics of skin cancer, surrounding microenvironment interactions, and regulation mechanisms to provide a broader perspective for new therapies’ development.

**Abstract:**

Skin cancer is one of the main types of cancer worldwide, and non-melanoma skin cancer (NMSC) is the most frequent within this group. Basal cell carcinoma (BCC) and squamous cell carcinoma (SCC) are the most common types. Multifactorial features are well-known for cancer development, and new hallmarks are gaining relevance. Genetics and epigenetic regulation play an essential role in cancer susceptibility and progression, as well as the variety of cells and molecules that interact in the tumor microenvironment. In this review, we provide an update on the genetic features of NMSC, candidate genes, and new therapies, considering diverse perspectives of skin carcinogenesis. The global health situation and the pandemic have been challenging for health care systems, especially in the diagnosis and treatment of patients with cancer. We provide innovative approaches to overcome the difficulties in the current clinical dynamics.

## 1. Introduction

Skin cancer is one of the most frequent types of cancer globally, being widespread among the Caucasian population [1]. The precise incidence of skin cancers is difficult to establish due to under-reporting, although prevention and diagnosis mechanisms are becoming more precise and effective [2,3]. Skin cancer basically includes malignant melanoma (MM) and non-melanoma skin cancer (NMSC), each with markedly different clinical outcomes [4]. Malignant melanoma represents the lowest percentage of all skin cancers, comprising only 5% of cases, and the diagnosis stage is essential for patients’ prognosis [5].

Regarding NMSC, despite not having a significant impact on the number of deaths from cancer, its incidence continues to increase with advancing age, and recent statistics showed more than one million cases per year [6]. Within this group, the major types are basal cell carcinoma (BCC) and squamous cell carcinoma (SCC); however, other types have been described with minor frequency [7]. BCC accounts for about 80% to 85% of NMSC cases, while SCC represents 15% to 20%, with a greater tendency to metastasize than BCC [8]. MM and NMSC differ in their tendency to metastasize, which places MM as the first cause of death among skin cancer patients [5].

Hallmarks of cancer are described as variable and complex, and risk factors for NMSC have been recognized early [9,10]. Among the known environmental causes, the major factor in the etiology of skin cancer is UV radiation (UVR), which may be classified in ranges based on wavelength as UVA1 (340–400 nm), UVA2 (320–340 nm), UVB (280–320 nm), and UVC (200–280 nm). Nevertheless, direct DNA injury is specifically associated with UVB, which can generate photoproducts such as pyrimidine dimers in the DNA of epidermal keratinocytes, affecting its stability and replication progression [11,12,13]. Given this fact, the presence of lesions in sun-exposed body areas can be explained. 

Similarly, internal processes such as the production of reactive oxygen species (ROS) can cause cell stress, and intracellular enzymes may be essential to protect their integrity [14]. Genetic factors play a crucial role in the development of cancer and specific mechanisms, genes, and tumor microenvironment features are involved. This review aims to update the genetic aspects that trigger NMSC, propose new candidate genes, and establish the relevance of the interacting cells and molecules in the tumor microenvironment. Additionally, we consider the current and challenging world health situation and new perspectives for diagnostic and therapeutic technologies.

## 2. Genetics of Non-Melanoma Skin Cancer and New Candidate Genes

Genetic aspects such as genomic stability and the presence of genetic variants that may change the expression patterns of tumor suppressor genes and oncogenes continue to be the focus of analysis to understand the origin of many types of cancer. DNA damage caused by UVR and its intensity is the principal hallmark of skin carcinomas [15]. Consistent with this, it can be estimated that one hour of exposure can generate 100,000 to 200,000 DNA lesions, affecting replication and transcription processes if they are not properly repaired [16,17]. Indeed, recent studies identified that NMSC lesions can acquire mutations that cause phenotypic changes towards more aggressive types. This plasticity of cancer cells allows them to escape from cellular regulation mechanisms [18].

Mhamdi-Ghodbani et al. [19] compared fibroblast, melanocytes, and keratinocytes isolated from human skin and their response to UVB radiation. Since keratinocytes are the main target in the epidermis for UVR, faster DNA repair, but no more efficient, was found in these cells. However, their repair capacity may not be sufficient to preserve genomic integrity, owing to the fact that they were not able to trigger apoptosis. Related to this circumstance, previous data collected from high-risk model SKH-1 hairless mice demonstrated the impact of UVR after being exposed to chronic-intermittent doses of solar-simulated radiation, and all the animals developed SCC carcinomas with histopathology spectra similar to humans. In addition, exome sequencing revealed mutations in tumor suppressor genes, which encode proteins that participate in cellular processes and signaling pathways [20].

**Figure 1:**
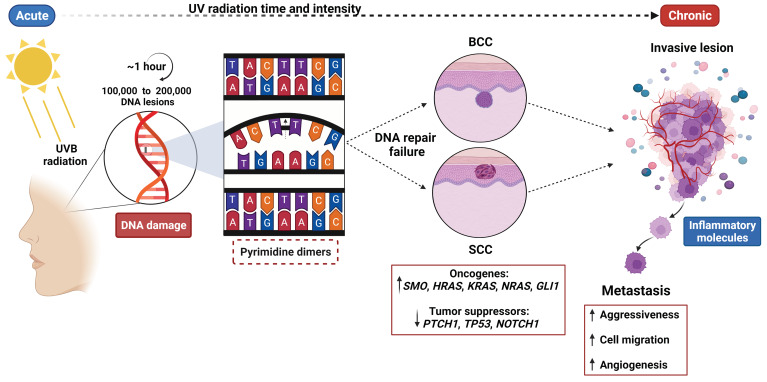
Environmental and genetic risk factors for non-melanoma skin cancer (NMSC). Environmental risk factors implicated in NMSC development are based on the intensity and time of UVB radiation upon exposed skin areas, and more importantly in the DNA of epidermal cells such as keratinocytes. These DNA lesions accumulate during UVB radiation exposure, which can be absorbed and generate DNA structural alterations such as cyclobutane pyrimidine dimers, causing the repair mechanisms to fail at some point and affecting replication and transcription cell processes. As a consequence, this interrupted gene function—especially with genes implicated in the regulation of cell proliferation, differentiation, and DNA repair—leads to skin cancer establishment, which will present higher rates of aggressiveness due to a progressive increase in inflammatory molecule activation, cell migration and angiogenesis. The present figure was created with BioRender.com, accessed on 4 May 2022. All icons, templates, and other original artwork appearing in the attached completed graphic are pursuant to BioRender’s Academic License Terms.

Repair mechanisms for DNA damage, such as DNA photolyase to remove covalent bonds between adjacent pyrimidines and nucleotide excision repair (NER) for cyclobutane pyrimidine dimers as a consequence of UVB radiation, are well-known (Figure 1) [17]. Candidate genes for UV response have been proposed. *SERPINB2* is known to regulate inflammation processes, apoptosis, and metastasis, but previous reports found a significant upregulation by UVR in several cell lines. This gene can regulate the removal of the NER complex from altered DNA, and contribute to tumor progression [21]. Moreover, UVR can induce the expression of the *ENTPD1* gene (also known as CD39) involved in purinergic signaling in skin-resident T cells. A higher gene expression was detected in T cells from SCC in contrast with immune normal cells from blood and skin. In addition, extracellular levels of adenosine were increased and its downregulation effect over gene coding for nucleosome assembly protein, a putative member of DNA repair machinery, led to the accumulation of DNA damage [22].

Environmental risk factors are well-known, and aging must be considered a personal risk factor for each individual, which can potentiate the effect of radiation exposure [23]. However, the early onset of NMSC may have substantial genetic implications [24]. Several genes and molecular mechanisms have been described to date. Alterations in the functioning of essential signaling pathways in cell growth control continue to drive carcinogenesis processes. Comparative studies have evaluated multiple microarray-based reports to determine possible genetic bases in the development of BCC and SCC. A consensus has been difficult to accomplish since these reports have overlapping genes and expression patterns in opposite directions [25]. The latest studies showed similar overlapping, but highlighted genes such as *CYFIP2*, *HOXB5*, *PTPN3*, *MARCKSL1*, *PTCH1*, and *CDC2* as diagnostic makers for the early detection of BCC [26]. Nonetheless, experimental validation of most of these reports is required.

Sonic Hedgehog signaling, a highly conserved developmental pathway in organogenesis from embryonic stages, and its role in tissue maintenance, regeneration, and repair, has been recognized for decades [13,27,28,29,30]. Verkouteren et al. [31] described that about 85% of sporadic BCC could display variants in genes implicated in Hedgehog signaling (*SHH*, *SMO*, *GLI1*, *GLI2*, *GL3*), in which loss of heterozygosity (LOH) is a common trait, although the profile of genetic variants in BCC is complex and variable. Additionally, a disrupted function of *TP53* gene has been established as the second most frequent cause of BCC, because of the lack of cell cycle arrest over DNA damage [15].

Through coding and non-coding somatic variant analysis in this type of NMSC in 191 patients, mutations in *PTCH1* and *TP53* genes were found in 58.6% and 31.4% of the cases, whereas variants in *TERT* and *DPH3* promoters were detected in 59.2% and 38.2%, respectively [32]. In contrast, a study cohort with a smaller sample of patients with sporadic BCC found *PTCH1* mutations in 72% of the cases, along with other genes such as *CSMD1* (63%), *TERT* promoter (58%), *DPH3* promoter (49%), *TP53* (46%), *NOTCH1* (44%), *DPP10* (35%) [33], representing the wide variability and cellular mechanisms involved in BCC pathogenesis.

Regarding SCC, earlier reports about *TP53* determined the role of this tumor suppressor gene in skin carcinogenesis mediated by UVB radiation, observing that exposed knockout mice developed this type of NMSC [34]. Recently, studies with whole-exome sequencing (WES) described the prevalence of oncogenic variants on this gene in 60% of the analyzed samples, in contrast to the adjacent UV-exposed epidermis [35]. Another important gene in skin development and differentiation is *NOTCH1*, for which missed or reduced signaling may lead to altered epidermal processes [36]. An orthotopic model of skin SCC showed the lack of expansion of cancer-associated fibroblasts (CAFs) due to *NOTCH1* gene silencing, without affecting the integrity of normal fibroblasts, and this may be significant for patients with a higher risk of skin carcinomas because of immune-suppressive therapies [37]. Similarly, Ras family proteins, which are encoded by *HRAS*, *KRAS*, and *NRAS* genes, are essential to cell proliferation, differentiation, and survival [38]. In a zebrafish embryonic simple epidermis model, it was proposed that KRas driver mutations can promote invasion, through the in vivo observation of cell migration, and simultaneously, disrupted p53 function was associated with cell survival but not with cell invasive capacity [39].

On the other hand, the immune system and its regulation are essential in NMSC emergence and tumor microenvironment establishment, as discussed below. Indeed, UVB radiation also has an impact on the immune response, which can induce immunosuppression, such as a reduction in Langerhans cell (from the skin immune system) antigen-presenting capacity, due to apoptosis and lymph node migration [40]. In addition, exposed macrophages may produce IL-10 cytokine as an immunosuppressive function and matrix metallopeptidases (MMPs) to maintain tumor progression [41]. The combination of UVB-induced ROS, DNA damage, cytokines, and chemokines release affects epidermal cells’ stability [42,43]. Alamartine et al. [44] reported that genetic variants in IL-10 could contribute to SCC susceptibility in organ transplant patients by favoring the escape of tumor cells from immune surveillance. The influence on BCC susceptibility and the clinical course has been also described [45,46]. Early comparative studies of the differential expression of chemokine receptors among pre-cancerous lesions, SCC, and BCC found the downregulation of *CCR6* and the upregulation of *CCR7* and *CXCR4* in invasive SCC, but not in BCC and actinic keratosis, consistently with the metastatic capacity of this type of NMSC [47]. However, as actinic keratosis is recognized as an SCC precursor, the accumulation of mutations may be determinant for their progression to malignant SCC [48].

From this genetic perspective, other immune molecules have been reported in association with NMSC risk. Programmed death-1 (PD-1), encoded by *PDCD1* gene, is expressed in several immune cells, such as T, B lymphocytes, and myeloid cells, having a negative effect on activated lymphocytes and limiting immune response. Genomic DNA analysis, obtained from 210 patients with BCC, found an allele-specific association (*p* < 0.02), suggesting that it may participate in BCC development; however, robust studies are required [49]. Additionally, cytotoxic T lymphocyte-associated protein 4 (CTLA4) has been investigated in another type of skin cancer, finding an inverse correlation between mRNA levels and promoter methylation, as should be expected. It is noteworthy that a low methylation level was correlated with a favorable treatment response, indicating that this pattern might be useful to predict the effect of specific antibodies targeting immune checkpoints such as PD-1 and CTLA4 [50]. Regarding genetic variants in *CTLA4* gene, it has been described in association with multiple lesions, and a higher expression is predominant in invasive lesion types [51,52].

Advances in bioinformatics have allowed access to large datasets. Weighted gene co-expression network analysis (WGCNA) comprises weighted correlation networks designed to recognize strong associations between key modules and genes, and even classify these genes within the modules. This study adds to the body of evidence that hub genes may be future biomarkers or therapeutic targets more accurate for the diagnosis and treatment of carcinomas [53]. Reports based on transcriptome sequencing and integrated bioinformatics have been able to identify and validate differentially expressed genes (DEGs) probably involved in SCC pathogenesis, although subsequent studies will be required to understand their mechanisms [54]. Despite that, there is still a lack of experimental evidence about most of the genes described by these methodologies. The investigation of candidate genes prevails, and the latest research regarding BCC and SCC is summarized in Table 1 and Table 2, respectively.

The reports presented above have a common limitation: sample size. We consider that further research is needed to accomplish more accurate conclusions and to elucidate these candidate genes’ implications in NMSC development. The majority of new variants reported have not been functionally characterized and there is still a lack of consensus about methodologies, experimental models, and the analysis of genetically distinct populations.

These findings may explain the heterogeneity of cancer, specifically in NMSC, where the transformation from an initially localized lesion to an invasive type requires genetic features. In this direction, previous reports identified that lesions with mixed histopathological characteristics may initially derive from a specific type and acquire variants that cause phenotypic changes towards more aggressive lesions types, and that this plasticity of cancer cells allows them to escape from some mechanism of cellular regulation [18,71]. From this scenario, the specific mechanisms involved in skin cancer development, and even cancer in general, are difficult to fully comprehend and classify, while they continue to be researched. Further investigation is required to establish the role of emerging genetic variants and, more importantly, their effect on candidate genes, as they are yet to become relevant.

## 3. Tumor Microenvironment, Cellular Components and Genetic Implications

**Figure 2:**
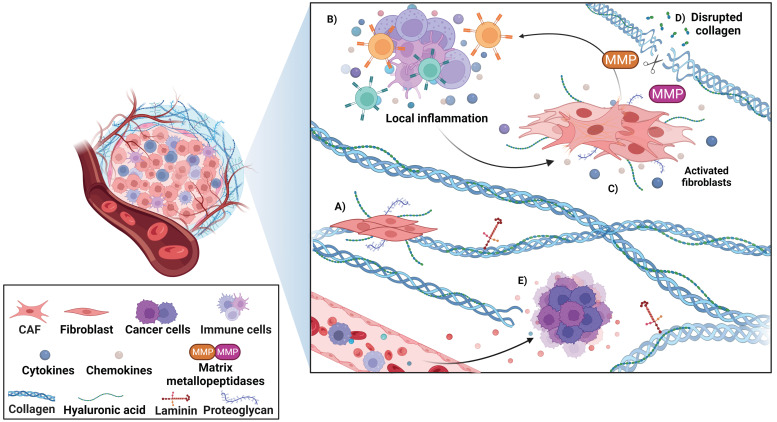
Tumor microenvironment in NMSC. The global tumor microenvironment is composed of different types of cells such as immune cells, endothelial cells (newly formed blood vessels), fibroblasts, and stromal proteins. (**A**) Fibroblasts are involved in the synthesis and degradation of extracellular matrix elements such as collagen, proteoglycans, and hyaluronic acid. (**B**) A mixture of immune cells and the production of cytokines and chemokines promote a local inflammation. (**C**) Activated fibroblasts can modulate the antitumoral response and maintain the inflammatory process. (**D**) The expression of matrix metallopeptidases (MMPs) permits the degradation of extracellular matrix and promotes tumor progression. (**E**) The angiogenesis process contributes to molecule exchange, providing nutrients and oxygen to cancer cells, and favoring their growing and invasive capacity. The present figure was created with BioRender.com, accessed on 4 May 2022. All icons, templates, and other original artwork appearing in the attached completed graphic are pursuant to BioRender’s Academic License Terms.

Cancer development does not depend only on external environmental and genetic features, but there are also a sequence of additional elements and mechanisms that encourage the establishment of neoplasms and their progression. In addition to cancer cells, there is a co-existent interaction among diverse types of cells such as immune cells, endothelial cells, fibroblasts, and stromal proteins in the tumor microenvironment, as illustrated in Figure 2. The presence of specific cell types may vary between primary and recurrent lesions, and even at different stages of treatment.

Lipson et al. [72] analyzed BCC tissue prior to immunotherapy application and found a mixture of CD4+, CD8+ T cells, and macrophages through the immunohistochemical technique, and PD-1 expression was detected in 50% of the present lymphocytes. Moreover, differences in the microenvironment immune response may be key to recurrence risk for BCC. Beksaç et al. [73] observed a lower presence of CD8+ T cells compared with CD4+ in primary tumors of patients who subsequently presented new lesions in the same anatomical area, proposing that a decreased antitumoral response represents a higher risk of tumor recurrence.

In fibroblasts, alterations due to UVB radiation are a classic hallmark of senescent cell accumulation [74]. Research on in vitro models of senescence with human dermal fibroblasts showed that these cells under UVB stress presented a dysregulated expression in genes involved with immune system modulation (*IL-1B*, *IL-6, CXCL1*, *CXCL2*), connective tissue promoters (*CTGF, ICAM1*), growth factors (*FGF2, TGFB1*), and matrix metallopeptidases (*MMP1*, *MMP3*). This secretory capacity is recognized as a senescence-associated secretory phenotype (SASP), and it is a presentative feature of CAFs [75].

CAFs are an abnormally activated type of fibroblast that act as synthetic machines of tumor components [76]. A key marker of this process is the expression of fibroblast activation protein (FAP), and its relation with poorer prognosis in several cancer types has been described [77]. Indeed, this protein has been reported in over 90% of epithelial carcinomas. Comparative studies of FAP expression between benign and malignant epithelial tumors found a stronger expression in fibroblasts neighboring the tumor, specifically in BCC and SCC [78]. Thus, the FAP protein might be utilized as a histological marker in the differentiation of specific BCC subtypes from benign cutaneous neoplasms [79]. In line with these findings, the endopeptidase activity of FAP may promote invasiveness in cutaneous cancers.

Given this fact, studies on matrix modification and the production of extracellular components in BCC tissue revealed that CAFs can produce chemokines (CCL17, CCL18, CCL22, CCL25, CXCL12) and cytokines (IL-6) that disrupt the local antitumoral response and redox balance through ROS production [80,81], as well as several types of collagens, proteoglycans, and hyaluronic acid, altering the equilibrium between synthesis and degradation of the matrix elements [82]. Indeed, comparative analysis among BCC, SCC, and melanoma described different protein expression patterns in CAFs, designated as phenotypes, suggesting that this may support tumor variability, which could be more complex than has been thought [83]. The clinical significance of this plasticity is still unclear, but these features can favor the idea that CAFs and chronic inflammation are an essential part of matrix remodeling and tumor microenvironment establishment, leading to skin carcinogenesis.

Furthermore, another homeostatic regulation process that must be considered is autophagy, which instead of promoting cell death contributes to their survival through protein, lipid, and organelle recycling, and this mechanism has been described in CAFs as well [84]. Consistent with the above, when CAFs experience metabolic stress, autophagy is activated and most of the protein fragments from catabolic processes can be released into the surrounding microenvironment, so the neighboring cells can uptake amino acids to produce energy when glucose is insufficient to maintain their metabolic activities, especially when higher growth rates demand several energy sources [85,86].

**Figure 3:**
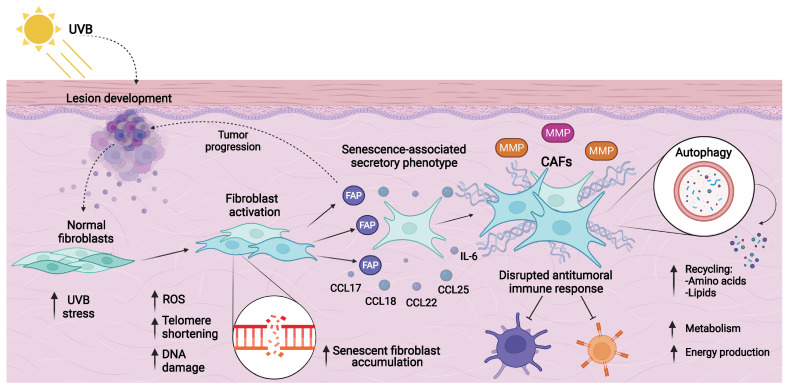
Fibroblast transformation to cancer-associated fibroblasts (CAFs) and their implication in the tumor microenvironment. Several elements and sequential phases are involved in the activation and transformation of fibroblasts into CAFs. CAFs acquire a distinct phenotype denominated as the senescence-associated secretory phenotype (SASP), which favors the accumulation of senescent cells. Fibroblast activation protein (FAP) is a key marker of fibroblast activation. Higher expression of matrix metallopeptidases promotes the establishment and progression of neoplasms, through extracellular matrix degradation, as previously mentioned. DNA damage due to UVB radiation is also a cause of fibroblast DNA integrity disruption, telomere shortening, and reactive oxygen species (ROS) production. Altered expression of immune system molecules, such as cytokines and chemokines, can interrupt the local antitumoral response and maintain this CAF activation process. In addition, autophagy produces amino acids and lipids fragments that provide energy sources to preserve an indispensable activated metabolism for cell transformation and migration. The present figure was created with BioRender.com, accessed on 4 May 2022. All icons, templates, and other original artwork appearing in the attached completed graphic are pursuant to BioRender’s Academic License Terms.

These characteristics could be considered isolated, but an important part of this effect is due to genetic variations in CAFs, where DNA damage and telomere shortening persist, and associated genes with skin cancer can induce cellular senescence. Increased *NOTCH1* function in these cells guarantees the upregulation of CAFs’ effector genes, which leads to tumor promotion (Figure 3) [37]. In BCC, preceding reports of isolated fibroblasts by laser capture microdissection allowed researchers to analyze gene expression, and upregulation in novel genes (*MGP*, *SFRP2*, *ANGPTL2*, and *PDGFRL*) was observed in independent samples [87]. These genes act as inhibitors of tissue calcification, soluble modulators of Wnt signaling and the formation of blood vessels, and proposed tumor suppressors, respectively. Highlighting the heterogeneity of cancer processes, the implication of diverse signaling pathways, cellular processes, and their regulation in malignant lesions’ evolution is established.

Moreover, the human keratinocyte carcinogenesis model showed the cell invasion capacity of SCC through activated H-Ras and transforming growth factor β (TGF-β) signaling pathways in fibroblasts, playing an essential role in fibroblast activation and higher expression of extracellular matrix elements. An upregulation in laminin-332 production, an important component of the skin basement membrane, leads to its accumulation, which correlates with tumor aggressiveness and poorer prognosis [88]. This approach is mainly related to SCC, given the knowledge of an increased metastatic capacity compared to BCC, where cell invasion rates are lower. However, CAFs’ implications in BCC progression could not be discarded. Altogether, CAFs’ differential gene expression provides a diversity of possibilities and demonstrates the variability of mechanisms that are necessary to change fibroblast behavior and promote cancer development.

In a broader sense, an important process for metastasis is angiogenesis, and the disturbance in the newly formed blood vessels due to the presence of fenestrations in their structure is crucial to molecule infiltration and accumulation [89]. Proteins that integrate the extracellular matrix are essential to cellular support, and their modification is required to permit cell communication and migration [90]. The comparison between the transcriptome of normal keratinocytes and SCC cell lines found a significant alteration in the transcripts of genes involved in cancer pathways, micro RNAs (miRNAs), and immune system signaling. In this respect, the secretome of cancer cells can influence the transcriptome of keratinocytes but not vice versa, leading to the fact that cancer cells can transform normal-healthy cells from the skin epithelium [91]. From this point of view, a differential proteomic analysis utilizing the mass spectrometry technique found an overlapping protein profile among precursor lesions and SCC. Simultaneously, increased protein levels were distinct for each type of lesion. Premalignant lesions presented altered proteins involved in cell cycle arrest, repair pathways, and apoptosis, whereas SCC showed a disrupted expression in proteins related to biological processes such as inflammation and angiogenesis, promoting the progression to invasive-type lesions [92].

In this regard, variations in the tumor secretome might signify an opportunity to implement new treatments. Targeting specific molecules and differentially expressed proteins related to lesions types or disease specific-stage may represent an opportunity to detain their progression towards more aggressive types, and even to metastasis in special cases. The modulation of microenvironment dynamics and constant remodeling could be an astute approach to enhancing therapies’ effectiveness, as well as supporting conventionally implemented therapies.

## 4. Epigenetic Regulation in Non-Melanoma Skin Cancer

As mentioned before, disrupted conditions of the tumor microenvironment are required for cancer establishment. Hanahan [9] described how cells surrounding the tumor may undergo epigenetic reprogramming, especially when they are recruited by molecules and growth factors that alter internal signaling pathways’ functioning. Likewise, external factors can cause broad changes in the epigenome and its response to cell and DNA damage. UVR’s effect upon human cells is well-known, and previous reports suggested that UVB radiation may have different influences on heterochromatin and euchromatin. This is because heterochromatin might affect major mutation incidence, and this could represent a type of protection over transcriptionally active DNA sequences. It has been proposed that chromatin with a peripheral location can absorb external energy and genetic harms to protect the nuclear core, representing a primary barrier to DNA damage, supporting the idea that epigenetic remodeling in skin cells can be transformed under malignancy states [93,94]. Besides this, DNA methylation is an important epigenetic regulatory element in gene expression. Aberrant methylation patterns are common in cancer cells, and the hypermethylation of promotors is related to tumor suppressor gene silencing [95]. Some genes may have an altered function due to epigenetic dysregulation rather than the presence of variants, and this is known to occur in genes associated with cell cycle regulation and DNA repair [96].

Other epigenetic mechanisms have emerged and continue to be elucidated. In cutaneous cancer, non-coding RNAs such as miRNAs and long non-coding RNAs (lncRNAs) are proposed to have a distinct role in cellular process regulation by targeting tumor suppressors, oncogenes, and transcription factors over the cancer course [96,97]. Mi et al. [98] evaluated miR-18a oncogenic activity in an epidermal cell line, finding an upregulation state in BCC compared with a control group. Furthermore, downregulation was related to the inhibition of cell proliferation, apoptosis, and autophagy activation through the Akt/mTOR signaling pathway. This outcome, from a well-characterized miRNA, may represent an effective therapy for BCC, and even for several types of cancer. Nonetheless, deeper research and the implementation of in vivo models are needed to establish their role in NMSC development.

On the other hand, high levels of attention to miRNAs regulation activity have been focused on SCC [99]. In this respect, another miRNA analyzed in human SCC cell lines is miR-130a, which was found to be downregulated in the SCC model but not in precancerous lesions, suggesting its role in the malignant phenotype of this type of NMSC. Moreover, overexpression of miR-130a may have a protective effect on invasive capacity through the inhibition of cell migration [100]. As previously mentioned, UVR has essential implications in NMSC development, and a recent study proved that UVR’s effect on miR-7-5p, miR-29a-3p, and miR-183-5p expression might vary based on the type of cell that was irradiated (primary and metastatic SCC, respectively) [101]. Under this circumstance, UVR may alter miRNA expression as well in the initial SCC lesion and possibly promote its progression to more aggressive types.

Epigenetic regulation has also been described in CAFs from the tumor microenvironment, where a remodeling interaction between miRNAs and extracellular matrix is present. Tissue analysis revealed differential expression of miRNAs in lesions and healthy tissue, and even in the comparison of superficial and deeper SCC. Higher expression of miR-21-5p and lower miR-1-3p was detected, when there was a higher immunoreactivity for matrix metallopeptidase 2 (MMP2) in CAFs. A downregulation of miR133a-3p shows its role in tumor progression, acting as a tumor suppressor [102]. Even though this study was conducted in oral SCC, we could not reject its extrapolation to cutaneous SCC, when keratinocytes alteration is common for both. Recently, the horizontal transference of miR-375 was also associated with CAFs presence due to fibroblast polarization, in a rare but more aggressive type of NMSC [103]. Consequently, epigenetic regulation also has a relevant role in matrix remodeling and fibroblast activation.

Novel molecules are becoming more relevant, and the possibility to improve future treatments is encouraging. Circular RNAs (circRNAs) are covalently closed circular RNA molecules, with post-transcriptional regulation activity and, therefore, roles in biological processes such as cell proliferation, metastasis, angiogenesis, and apoptosis [104]. This can be explained by the fact that circRNAs can act as miRNAs sponges due to complementary sites, and therefore block miRNAs’ activities [105]. In BCC, novel circ_0005795 was analyzed in tissue lesions from patients and BCC cell lines, finding a significantly increased expression in both models, and simultaneously, knockdown of this circRNA reduced cell viability, highlighting its oncogenic role in NMSC [106]. In contrast, Li et al. [107] found significantly higher expression of circ_0067772 in SCC tissue when compared with normal adjacent skin, and knockdown through interference RNA revealed a decreased cell proliferation and migration. The authors claimed that this is the first report about circ_0067772 roles in SCC cell proliferation and maybe metastasis, being an optimistic target for future treatment improvement. The application of regulatory RNA molecules may be meaningful in therapy-resistant cancer types. Studies continue to accumulate evidence, and non-coding RNAs reported in recent years for BCC and SCC are presented in Table 3 and Table 4, respectively. However, more research is required to elucidate further mechanisms.

## 5. Perspectives: About Known Mechanism and New Alternatives

Taking into account the fact that the characteristics of cancer can be heterogeneous and widely variable [9], new alternatives are being raised for patients who are not benefitted by surgical management due to the presence of highly recurrent lesions, or even metastatic states. Although important advances have been achieved in BCC via targeted treatments that inhibit Sonic Hedgehog signaling, an inhibitor of immune checkpoints such as cemiplimab (anti-PD-1/PD-L1) may have promising outcomes [116,117]. A phase 2 trial demonstrated for the first time the significant antitumor activity of systemic therapy with cemiplimab in locally advanced BCC, focused on patients who were previously intolerant to Sonic Hedgehog inhibitor therapies [118]. Nonetheless, longer periods of follow-up will be necessary to obtain a better understanding of clinical response. In this respect, in more severe types of cancer, a phase 1 multicenter study performed the characterization of the lymphocyte-activation gene 3 (LAG-3) inhibitor, ieramilimab. LAG-3 is an inhibitory immunoreceptor expressed in several immune cells, and its high-affinity ligand is the major histocompatibility complex class II (MHC-II). Blocking LAG-3 has been demonstrated to improve cytotoxic T-lymphocyte proliferation, and for this study, a tolerable response was observed as a monotherapy and in combination with anti-PD-1 spartalizumab [119], highlighting the importance of inhibitor of immune checkpoint therapies in metastatic cancers.

When it comes to understanding tumor behavior, it is necessary to analyze tumors as complete pieces of machinery that can maintain their progression. Since autophagy and extracellular matrix modification and CAF activity are involved in tumor advance, new therapies are becoming potent tools for cancer control. As described by Zhang et al. [120], topical 5-aminolevulinic acid photodynamic therapy (ALA-PTD) may be a good option, since it does not present drug resistance and allows localized treatment. This study demonstrated that suppressed proliferation and induced apoptosis were achieved in SCC cells by the combination of chemotherapy agents, an autophagy inhibitor, and PTD simultaneously, suggesting a promising treatment strategy. Moreover, Li et al. [121] described a reversible activation of CAFs when ALA-PTD was applied to transformed fibroblasts previously co-cultured with SCC cells, due to a decreased expression of markers such as FAP and a reduction in their migratory capacity. This may lead to the idea that PTD could be used in invasive-type lesions and not only in superficial types. Previous studies on fibroblast sensibility to PTD in patients with genetic diseases with skin cancer predisposition highlighted its relevance in target epithelial elements as a treatment alternative [122]. However, a recent report on CAFs generated by co-cultures of normal human fibroblasts with cancer cells found radioresistance patterns through a decrease in micronucleus formation, a chromosomal damage hallmark due to DNA breaks, and this may signify that the survival of CAFs through radiation therapies continues to support tumor growth [123].

Since epigenetics is becoming relevant, a case–control study analyzed DNA methylome and created a DNA methylation-based risk identification index in European women for breast cancer, based on the hypothesis that systemic epigenetic programming may be determinant for cancer development [124]. Therefore, this type of evaluation cannot be dismissed for skin cancer, or even for any type of cancer, considering the importance of genetic and epigenetic aspects previously mentioned. The regulation of gene expression, especially for genes with tumor suppressor activity, might be an accurate approach for new diagnostic and prevention methodologies in different populations.

Last but not least, the transition to new technologies is becoming increasingly urgent, and health professionals will gain powerful diagnostic tools [125]. Artificial intelligence (AI) is based on computer algorithms, and its derivatives can recognize patterns of images, which will be useful for dermatopathologists. Nevertheless, the current and conventional clinical environment may not be prepared to fully introduce this technology into practice [126]. These advances are not focused on denying physicians’ expertise or even trying to replace them, but future implementation will be a supporting feature to improve diagnosis and clinical procedures. The development of image recognition apps may have a positive impact on early diagnostics, considering the current limitations in proper health care.

## 6. Skin Cancer and the COVID-19 Pandemic

The COVID-19 pandemic changed the global clinical dynamics, and the real impact around the world is still unknown. Delays in cancer diagnosis and treatment due to the collapse and reduced availability of health systems may cause an apparent reduction in cancer incidence, followed by higher mortality as a consequence of the advanced stages of the disease at diagnosis [6,127]. The comparison of similar periods before and after COVID-19 showed a significant delay in overall cancer diagnoses and specifically, in skin cancer, a 69% decrease in diagnoses was observed in the European population [128].

SARS-CoV-2 virus infection in oncological patients needs to be considered to establish appropriate management strategies and thus avoid disease complications. The approach introduced by Baumann et al. [129] states that postponing skin cancer treatments may lead to higher mortality risk due to disease progression. Lethality for MM represents more than 75% of all deaths related to skin cancer, and for NMSC cases, the American Cancer Society estimates about 2000 deaths in the USA each year from BCC and SCC. Nonetheless, mortality is mostly related to SCC patients, who can experience metastases in 4% of the cases [14,130,131]. There are no reports of treatment delay in BCC and mortality risk. 

In addition, Tagliaferri et al. [132] proposed that radiotherapy should be considered in older patients with advanced SCC or BCC, particularly when surgical procedures are not achievable. In this respect, diagnoses and treatment retardation may negatively affect health costs [133,134]. Recommended strategies regarding the risk classification of cancer patients can be applied to NMSC, which could be categorized as low-risk based on progression due to treatment delay [135].

**Figure 4:**
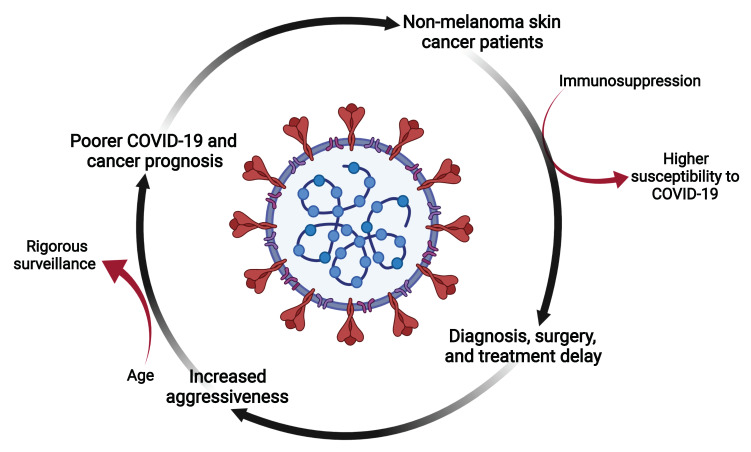
Non-melanoma skin cancer and COVID-19. The COVID-19 pandemic and the related hospital crisis caused health care saturation, and proper attention to patients with special needs was interrupted for more than a year. For patients with NMSC, and even for all types of cancer, a delay in diagnosis and treatments was common. In addition, cancer patients have an increased risk for COVID-19 complications due to immunosuppressive states, mainly caused by debilitating therapies, and the disease itself. This challenging situation may signify an opportunity to develop new strategies to treat patients and overcome current health conditions. The present figure was created with BioRender.com, accessed on 4 May 2022. All icons, templates, and other original artwork appearing in the attached completed graphic are pursuant to BioRender’s Academic License Terms.

On the other hand, skin cancer can be a debilitating disease and immunosuppression is common among these patients. Due to this fact, susceptibility to infections such as COVID-19 can be higher compared to the general population [136,137], and tumors might be more aggressive and recurrent [138]. A prospective cohort study in China described a poorer prognosis for COVID-19 in cancer patients and recommended an intentional postponement of chemotherapy or surgery, greater personal protection for patients or cancer survivors and, finally, more rigorous surveillance or treatment in cases where cancer patients are infected. These observations are fundamental for endemic areas and for patients with advanced age [139]. The screening of affected individuals will be crucial to recognize high-risk patients, and the implementation of individual strategies must be considered to improve monitoring and even to determine the intensity of the treatments to be implemented (Figure 4).

## 7. Conclusions

The biology of cancer is complex, and NMSC is the most frequent type of skin cancer worldwide. Environmental risk factors such as UVB radiation from sunlight can directly damage the DNA of epidermal cells. Genetic features and failure in DNA repair mechanisms continue to be a determinant of cancer susceptibility. Up to now, beyond the already known risk factors, it is difficult to achieve a consensus for NMSC triggers, due to the variability of involved components. However, these features may represent an opportunity to advance in new therapies’ development that not only focus on removing cancer cells, as the source of tumor origin, but also on a sequence of immunological, genetic, epigenetic, and stromal events. Recognizing tumors as pieces of a machine for growth factors, inflammatory molecules, and extracellular matrix protein production, functioning together for cancer establishment and progression, may be crucial for successful cancer treatment. Finally, since the appearance of COVID-19, health systems have faced unimaginable challenges, and patients with chronic diseases suffered the consequences of late diagnosis and treatment, leading to the opportunity for new technologies’ application to improve the prognosis of skin cancer patients.

## Figures and Tables

**Table 1:** Candidate genes for BCC.

Genes	Methods	Findings	Reference
**Tumor suppressor**			
***LRP1B***	Tissue	Most frequently mutated gene in 50% of analyzed samples Candidate BCC driver gene in Korean patients	[55]
**Immune system**			
***IL-6***	Peripheral blood	Variants in IL-6 are associated with higher risk of BCC	[56]
***SOCS3***	Cell line Murine model	Use of peptides mimicking the action of SOCS3 may inhibit the proliferative effect of IL-22 upon transformed keratinocytes	[57]
**Extracellular matrix degradation**			
***MMP1*** ***MMP3***	Tissue	Matrix metallopeptidases are involved in tumor progression through extracellular matrix degradation	[58]
**Cell processes**			
***DDX5***	Tissue Cell line	Gene knockdown increased apoptosis and suppressed migration and invasion of BCC cells	[59]
***GREM1***	Tissue	Invasive cancer cells may induce *GREM1* expression in fibroblasts and BCC subtypes can be determinants for protein expression levels	[60]
***PTPN14***	Large-scale next generation sequence data	BCC predisposition gene through germline loss of function variants	[61]
**Protein crosslinking**			
***TGM3***	Tissue	Overexpression of *TGM3* in BCC tissue New potential specific marker for BCC	[62]

*LRP1B*: LDL receptor-related protein 1B; *IL-6*: interleukin 6; *SOCS3*: suppressor of cytokine signaling 3; *MMP1*: matrix metallopeptidase 1; *MMP3*: matrix metallopeptidase 3; *DDX5*: DEAD-box helicase 5; *GREM1*: gremlin 1; *PTPN14*: protein tyrosine phosphatase non-receptor type 14; *TGM3*: transglutaminase 3.

**Table 2:** Candidate genes for SCC.

Genes	Methods	Findings	Reference
**Cellular communication**			
***ALK***	Murine model	Variants driving constitutively active function trigger oncogenic signaling for SCC	[63]
**Cell cycle**			
***CDC20***	Cell line	Inhibition of SCC proliferation through gene silencing. Novel target for diagnosis and treatment	[64]
***CDK1***	Bioinformatics analysis research	Higher expression in SCC Gene expression analysis provides a predictive tool in tumor development and progression understanding	[65]
**Immune system**			
***C1R***	Murine model Cell line	Role of C1r in SCC tumor growth and invasion by increasing matrix metallopeptidases production	[66]
***C3***	Cell line	Tumorgenic effect of C3 in SCC during chronic inflammation in the skin	[67]
***SOCS3***	Cell line Murine model	Use of peptides mimicking the action of SOCS3 may inhibit the proliferative effect of IL-22 upon transformed keratinocytes	[57]
**Cell proliferation and differentiation**			
***HOXB7***	Cell line	Gene knockdown accelerates apoptosis, suppresses cell migration and tumor progression	[68]
**Tumor suppressor**			
***LRP1B***	Tissue	Increased expression in metastatic SCC Possible predictive value for immunotherapy response	[69]
**Transcription factor**			
***TCF4***	Cell line	Interference of *TCF4* expression played an important tumor-repressive role in SCC by the inhibition of signaling pathways activation	[70]

*ALK*: anaplastic lymphoma receptor kinase; *CDC20*: cell division cycle 20; *CDK1*: cyclin dependent kinase 1; *C1R*: complement C1r; *C3*: complement C3; *SOCS3*: suppressor of cytokine signaling 3; *HOXB7*: homeobox B7; *LRP1B*: LDL receptor-related protein 1B; *TCF4*: transcription factor 4.

**Table 3:** Non-coding RNAs implicated in BCC.

Non-Coding RNA	Samples and Methods	Status	Proposed Role	Limitations	Reference
***miRNAs***					
**miR-34a**	86 Serum	Downregulation	Tumor suppressor	Small sample size	[108]
**miRNA-451a**	22 Tissue Murine model	Downregulation	Tumor suppressor	Small sample size	[109]
***circRNAs***					
**Circ_NCKAP1**	3 Tissue Cell line	Upregulated	Tumor promoter	Limited sample size Lack of in vivo model	[110]

**Table 4:** Non-coding RNAs implicated in SCC.

Non-Coding RNA	Samples and Methods	Status	Proposed Role	Limitations	Reference
***miRNAs***					
**miR-10b**	- Cell line	Upregulated	Tumor promoter	Lack of evidence on animal models	[111]
***lncRNAs***					
**RP11-493L12.5**	28 Tissue	Upregulated	Unknown	Small sample size	[112]
**KB-1410C5.3/lnc-GRHL2**		Downregulated	Unknown		
***circRNAs***					
**circ_0070934**	38 Tissue Cell line	Upregulated	Tumor promoter	Downstream targets of miRNAs sponged by this circRNA are still unknown in SCC	[113]
	- Cell line	Upregulated	Tumor promoter	Tissue from patients is needed to asses clinical value	[114]
**circ_EPSTI**	28 Tissue	Upregulated	Unknown	Small sample size	[112]
**circ_IFFO2**		Downregulated	Tumor promoter		
**circ_0001360**	3 Tissue	Downregulated	Tumor suppressor	Small sample size	[115]
